# Pre-Procedural Use of Levosimendan in High-Risk ACS-PCI Patients with Reduced Left Ventricle Ejection Fraction—Short-Term Outcomes

**DOI:** 10.3390/jcm14082761

**Published:** 2025-04-17

**Authors:** Karol Turkiewicz, Jan Jakub Kulczycki, Piotr Rola, Szymon Włodarczak, Mateusz Barycki, Piotr Włodarczak, Łukasz Furtan, Paweł Kozak, Adrian Doroszko, Waldemar Banasiak, Maciej Lesiak, Adrian Włodarczak

**Affiliations:** 1 Department of Cardiology, The Copper Health Centre (MCZ), 59-301 Lubin, Poland; karolt777@gmail.com (K.T.); jan.jakub.kulczycki@gmail.com (J.J.K.); wlodarczak.szy@gmail.com (S.W.); wlodarczak.piotr@outlook.com (P.W.); pawelkozakmd@gmail.com (P.K.); wlodarczak.adrian@gmail.com (A.W.); 2 Faculty of Medicine, Wroclaw University of Science and Technology, 50-981 Wroclaw, Poland; adrian.doroszko@gmail.com (A.D.); waldemar.banasiak@pwr.edu.pl (W.B.); 3 Department of Cardiology, Provincial Specialized Hospital, 59-220 Legnica, Poland; mateusz.barycki@gmail.com (M.B.); lukas.furtan@gmail.com (Ł.F.); 4 Department of Cardiology, Center for Heart Diseases, 4th Military Hospital, Faculty of Medicine, Wroclaw University of Science and Technology, 50-981 Wroclaw, Poland; 5 1st Department of Cardiology, University of Medical Sciences, 61-848 Poznan, Poland; maciej.lesiak@skpp.edu.pl

**Keywords:** acute coronary syndrome, levosimendan, high-risk percutaneous coronary intervention, PCI, acute coronary syndrome (ACS)

## Abstract

**Background/Objectives:** Current evidence suggests that levosimendan may have a beneficial effect in the treatment of acute heart failure (AHF) or cardiogenic shock following primary percutaneous coronary intervention (PCI). However, there is a paucity of data on the use of levosimendan prior to PCI. Therefore, our pilot study aimed to assess the short-term prognosis of a new therapeutic protocol involving preprocedural infusion of levosimendan in patients with reduced left ventricular ejection fraction undergoing high-risk PCI for acute coronary syndrome (ACS). **Methods:** The study is a retrospective observational study, and the population includes all subjects who received levosimendan infusion prior to high-risk PCI for ACS. Subjects requiring urgent revascularization (cardiogenic shock, cardiac arrest) or with mechanical complications of ACS were excluded. **Results:** The study cohort consisted of 90 subjects, predominantly men (91.1%) with significantly reduced left ventricular function (28.7% (12)) and advanced coronary artery disease, mean SYNTAX Score 25.8 (19.3–33). During in-hospital follow-up, we observed 2 primary outcomes—death. The major adverse cardiac and cerebrovascular events (MACCE) rate was 7.8%. Two clinical adverse events that did not lead to discontinuation were observed during the in-hospital period. Both were related to hypotension. **Conclusions:** In short-term observation, novel therapeutic approach in the management of high-risk PCI in ACS patients—pre-procedural levosimendan—was a relatively safe approach. No significant adverse events were reported.

## 1. Introduction

Despite the undeniable advances in pharmacological and interventional therapy, coronary artery disease (CAD) remains the leading cause of death worldwide, with a particularly elevated mortality rate in patients with acute coronary syndrome (ACS). The primary determinant of successful clinical outcomes in the treatment of patients with acute coronary syndrome (ACS) is the achievement of complete revascularization. Although the current guidelines recommend coronary artery bypass (CABG) surgery as the preferred revascularization option for the subpopulation of patients with advanced coronary artery disease (CAD), in routine daily practice, percutaneous coronary intervention (PCI) remains the definitive revascularization strategy due to the aging of the population and the high burden of comorbidity. To improve clinical outcomes and augment the safety in the high-risk PCI-ACS cohort, novel devices and therapeutic strategies have been introduced into clinical practice.

Levosimendan is a novel therapeutic molecule with a unique mechanism of action that was initially approved for the stabilization of patients with acute decompensated heart failure. It is postulated that this novel cardiovascular pharmaceutical agent exerts a cardioprotective effect, in addition to providing an inotropic effect. Furthermore, it has been demonstrated to improve cardiac contractility by increasing calcium sensitivity and promoting vasodilation through the opening of adenosine triphosphate-dependent potassium channels. Specifically, three main pharmacological actions have been identified as the principal mechanisms of action. First, there is selective binding to Ca^2+^-saturated cardiac troponin C. Second, there is the opening of ATP-sensitive potassium (KATP) channels in the vasculature. Finally, there is the opening of KATP channels in the mitochondria. Unlike other inotropic drugs used to treat acute HF, levosimendan does not increase intracellular calcium levels, which means that cardiac myocytes consume less energy (measured in ATP usage), resulting in a significantly reduced total oxygen consumption compared to other inotropic drugs [1]. This unique pharmacology justifies its use in a wide range of clinical applications, such as cardiogenic shock, various types of cardiomyopathy, pulmonary hypertension, cardiac surgery, emergency care, or even oncology treatment protocols [1,2,3].

Current evidence suggests that levosimendan may have a beneficial effect on the treatment of acute congestive heart failure (CHF) or cardiogenic shock following primary percutaneous coronary intervention (PCI) [4,5,6]. In addition, evidence suggests a clinical benefit of adding levosimendan to the preoperative treatment protocol in patients undergoing open-heart surgery [7,8]. However, there is a paucity of data regarding the use of levosimendan prior to PCI [9]. Furthermore, it is important to consider the potential benefits of myocardial preconditioning in the context of levosimendan, given its established renal and cardioprotective effects [10]. This may provide additional unrecognized clinical benefits, particularly in high-risk PCI cases, which have thus far been overlooked.

This study aims to evaluate the short-term prognosis of a new therapeutic protocol involving pre-procedural infusion of levosimendan in patients with reduced left ventricular ejection fraction undergoing high-risk percutaneous coronary intervention for acute coronary syndrome (ACS-PCI).

## 2. Materials and Methods

### 2.1. Study Population

The study is of a retrospective observational nature, and the population included all subsequent subjects who underwent high-risk percutaneous coronary intervention (PCI) in the context of acute coronary syndrome (ACS) between May 2019 and March 2024 at two high-volume cooperative cardiology units in the Lower Silesia Region of Poland. The study population was carefully selected from among all 5846 ACS cases treated in both centers during the specified study period.

The following criteria were employed to determine which subject would be included in the study cohort:

(1) Initial diagnosis of ACS;
(2) Revascularization achieved with percutaneous coronary intervention (PCI);
(3) Pre-PCI infusion of levosimendan
(4) Presence of anatomical features of CAD fulfilling the criteria of high-risk PCI
(5) Presence of significantly impaired LV function (ejection fraction equal to or below 35%).

The qualification for PCI was made either based on a judgment made by the Heart Team (of all ACS patients, 624 were referred to cooperative cardiac surgery department as potential CABG candidates) or a particular clinical indication, including but not limited to ongoing ischemia, lack of will for alternative treatment options, and the presence of CAD suitable for percutaneous coronary intervention (PCI) in accordance with ESC/ESH recommendations. All patients were provided with comprehensive information regarding the available therapeutic options and the potential risks associated PCI prior to providing written informed consent for the procedure. The study was approved by the local ethics committee (Lower Silesian Medical Chamber, ref.7/BODB/2021 date of approval–09.06.2021). Additionally, patients who completed the standard 12-month follow-up period were invited to sign an informed consent form to participate in a more extensive clinical evaluation. The results of these observations are to be published at a future date.

**Figure 1:**
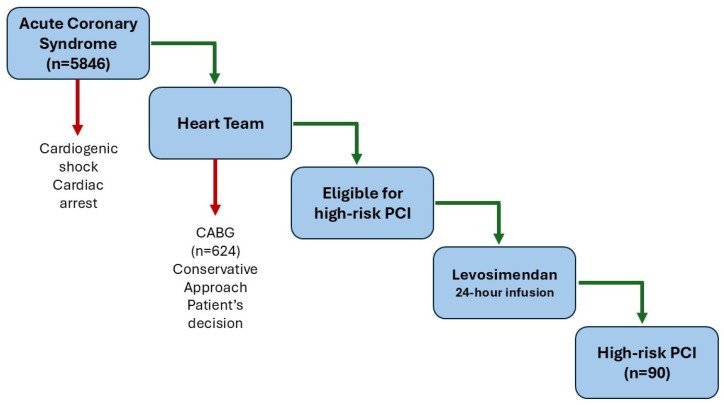
Study flow chart.

High-risk PCI was defined according to the generally accepted consensus [11] and included subjects with left main disease, multivessel disease involving the proximal LAD, intervention of the last patent vessel, or complex intervention in patients with severely reduced left ventricular (LV) function (35% or less). The diagnosis of ACS was made according to the current ESC/ESH recommendations. Patients with the following exclusion criteria were not included: patients with cardiogenic shock (defined according to consensus [12]) requiring urgent revascularization or cardiac arrest on hospital admission. Patients with mechanical complications of ACS (e.g., ventricular septal defect, left ventricular thrombus) were also excluded from the study. All subjects in the study received a 24-h infusion of levosimendan (cumulative drug dose: 12.5 mg per subject) at least 24 h before undergoing high-risk PCI. The initial pre-load dose of levosimendan was not used in this study. The study flow chart is shown in Figure 1.

### 2.2. Study Endpoints

The Academic Research Consortium definitions were used to assess all study endpoints [13]. Basic study follow-up focused on the time of discharge and the 30 days after discharge. Clinical follow-up was conducted by medical professionals, in person or by telephone contact. The primary endpoint was mortality. The secondary endpoint was a composite of major adverse cardiac and cerebrovascular outcomes (MACCE), including mortality, acute MI, repeat revascularization, and stroke.

The remaining study outcomes focused on in-hospital adverse events related to therapy, including those directly related to levosimendan infusion (e.g., clinically relevant ventricular arrhythmias, infusion-related hypotension leading to drug aversion and allergic reactions) or related to PCI (e.g., coronary perforation, slow or no reflow, ventricular arrhythmias). In addition, cases of acute decompensated heart failure (ADHF) that were considered clinically relevant were reported.

In-hospital clinically relevant ADHF episodes were defined as worsening symptoms requiring additional prolongation of hospitalization or intensified medical treatment, including diuretics, vasodilators, inotropes and/or left ventricular assist device implantation. Regarding the post-discharge period, the clinical relevance of ADHF episodes was determined by the need for hospitalization. In addition, the occurrence of post-procedural acute kidney injury (AKI), characterized by an increase in creatinine of ≥0.3 mg/dL (≥26.5 μmol/L) or ≥1.5 times baseline [14], and bleeding events (defined as bleeding fulfilling criteria 2–5 of the Bleeding Academic Research Consortium [BARC] scale) [15] was documented.

### 2.3. Statistical Analysis

Statistical analysis was performed using the R language. The *p*-value was considered statistically significant at the 0.05 level. Normality of the distribution was determined using the Shapiro–Wilk test. The results are presented as the mean with standard deviation (SD) or median with interquartile range (Q1–Q3), depending on the distribution. Categorical data were analyzed using McNemar’s test, while continuous data were analyzed using Student’s paired *t*-test or Wilcoxon paired signed rank test, depending on the results of the Shapiro–Wilks test for normality.

## 3. Results

The study cohort was predominantly male (91.1%), with a mean age of 69.5 years. The majority of patients were admitted to the hospital for non-ST elevation ACS (91.1%), with only 8 patients having a primary diagnosis of ST elevation myocardial infarction (STEMI). The study cohort had a high prevalence of classical cardiovascular risk factors, with hypertension (83.3%), hyperlipidemia (76.6%), and type 2 diabetes (43.3%) being the most common. All patients had advanced CAD with a mean SYNTAX Score of 25.8 (19.3–33) points. The average hospital stay was 11.5 (5.9) days. During hospitalization, a significant increase in LV function was observed when comparing the initial echocardiographic assessment with the discharge assessment (28.7 (12.0) vs. 34.4 (11.3), *p* < 0.01). In contrast, there was a non-significant increase in creatinine levels between admission and discharge (96.6 (76–102.2) vs. 99.1 (79.5–111); *p* = 0.182). A detailed analysis of pharmacotherapy in the study cohort showed that the vast majority of patients were receiving dual antiplatelet therapy with acetylsalicylic acid (96.6%) and clopidogrel (72.2%). The use of novel heart failure-specific agents was comparatively low, with only 40% of patients receiving sodium/glucose co-transporter 2 inhibitors (SGLT-2 inhibitors) and 21.1% receiving dual-acting angiotensin receptor-neprilysin inhibitors (ARNI). All demographic and clinical characteristics are summarized in Table 1.

Data regarding periprocedural characteristics are presented in Table 2. The data show that more than half of the patients underwent left main intervention (53.3%). The mean SYNTAX Score was 25.8 (19.3–33) points. The vast majority of study subjects received multivessel PCI, resulting in high contrast use—206.8 mL (130.7–265.5) and radiation dose—1128 mGy (588–2085). Regarding periprocedural complications of PCI, three clinically relevant periprocedural myocardial infarctions (MI) were identified. Two of these were attributed to acute occlusion of significant side branches, and one to prolonged slow-flow phenomena. In addition, three patients experienced significant arrhythmias, including two sustained ventricular tachycardias and one pulseless electrical activity (PEA). Additionally, one case of distal wire perforation was managed conservatively.

During the in-hospital follow-up, we observed 2 primary outcomes: death. The first case concerns a 67-year-old man with a high comorbidity burden of extremely advanced CAD (chronic total occlusion of RCA, Cx, and significant stenosis of the LM and LAD) with co-existing advanced HF LEVF—20%. The patient successfully underwent PCI and on day 9 of hospitalization, shortly before discharge day, without any prodromal symptoms, experienced advanced A-V block—despite prolonged ALS and implementation of an endocavitary electrode, the patient died. A secondary death was observed in an 82-year-old man with severe chronic obstructive pulmonary disease (COPD) who was receiving home oxygen therapy. The patient underwent successful percutaneous coronary intervention (PCI) but subsequently died of advanced pneumonia, leading to complete respiratory failure.

In terms of secondary study outcomes in the study cohort, we observed a 7.8% rate of MACCE.

Two clinical adverse events connected to levosimendan infusions were observed in this study cohort. These events were associated with hypotension. In both case subjects, a small dose of norepinephrine was required, but the infusion was not stopped, and the patient received the full 12.5 mg dose. Furthermore, despite the relatively low mean LVEF (28.7% (12.0)) observed in the study cohort, only three subjects experienced clinically relevant acute heart failure following successful infusion of levosimendan.

A relatively high incidence of periprocedural AKI (18.8%) was observed, but no subjects in the study required renal replacement therapy. During the in-hospital phase, five subjects experienced clinically relevant bleeding events. Three of these, were related to PCI access sites, while two were gastrointestinal bleeding events.

Analysis of the 1-month post-discharge period did not reveal any additional primary study endpoints. Three additional major adverse cardiac events (MACEs) were observed in the study cohort, all related to MI. Two of these were related to target lesion revascularization, and one patient experienced an MI in a previously untreated vessel. Additionally, one subject was hospitalized due to ADHF during this period. Study results are shown in Table 3.

## 4. Discussion

To the best of our knowledge, this is one of the first studies published thus far that addresses the use of a levosimendan infusion as an adjuvant treatment prior to high-risk PCI in subjects with ACS and severe ischemic dysfunction of the left ventricle. The primary findings of our preliminary study are as follows.

1. The administration of levosimendan infusion prior to high-risk ACS-PCI was a relatively safe approach. A negligible number (2.2%) of adverse effects were observed, which were clinically irrelevant and did not lead to discontinuation of the drug.
2. Despite the significant improvement in LV function observed in the study cohort at discharge compared to admission, large, randomized trials are needed to fully evaluate the potential clinical benefits of a pre-PCI levosimendan infusion in course of ACS, with particular interest in the impact on renal and LV function.

Patients suffering from acute coronary syndrome and complex multivessel diseases with co-existing severely impaired left ventricles remain one of the most challenging clinical subsets in modern cardiology. The co-existence of advanced heart failure (HF) and multivessel coronary artery disease (CAD) poses a considerable risk in relation to the outcomes of all revascularization procedures [16,17]. Even though current guidelines [18] advocate coronary artery bypass surgery (CABG) as the optimal therapy, in routine clinical practice, due to the high burden of comorbidities and the increasing age of the population, the number of PCIs in this subpopulation is constantly growing. While publication of the results of the RE-VIVED-BCIS2 [19] study has prompted a debate regarding the purposefulness of PCI in patients with stable CAD and severe ischemic left ventricular dysfunction, but achieving complete revascularization remains the gold standard for subjects with ACS [18]. The enhancement of outcomes for patients with severely reduced LV function undergoing PCI, particularly those with ACS, is a salient clinical concern, especially in light of the fact that extant data appear to support the use of alternative revascularization techniques in patients with advanced multivessel CAD [20,21].

Recently published data indicate that enhancement in LV function subsequent to PCI serves as an independent marker of improvement in the general prognosis of patients with CAD and co-existing ischemic heart failure, irrespective of the initial severity of heart systolic function impairment [22,23]. As demonstrated in our study, the improvement observed in postprocedural LVEF (5.7%) appears to reach levels that have been claimed to be prognostic. he enhancement in contractile function observed in this study is presumably attributable to successful PCI; nevertheless, further investigation is required in the form of randomized trials with a control group to fully evaluate the beneficial effect of levosimendan on cardiac contractile function. The relationship between the improvement in postprocedural LVEF and favorable long-term clinical outcomes is most clearly demonstrated in subjects analogous to the study cohort, namely the ACS subset [24].

Despite the suggestion of several factors as potential predictors of LVEF improvement after PCI, there is still a paucity of well-validated reliable markers.

Furthermore, given the absence of a definitive understanding of the precise mechanisms underlying these improvements, therapeutic interventions aimed at this objective are more likely to be the focus of scientific research rather than being incorporated into standard clinical practice. Conversely, several technical aspects strictly related to PCI have the capacity to influence long-term outcomes following percutaneous revascularization. These aspects include adequate lesion preparation, the utilization of intravascular imaging, and optimal post-procedural stent optimization [25,26,27,28,29,30].

However, there is an absence of data that is pivotal to the formulation of strategies that will lead to improvements in patient outcomes that are not directly related to the quality of PCI intervention in vessels. A recently published study suggests that the routine use of a mechanical circulation support device, namely the microaxial flow pump Impella CP, has the potential to positively impact the outcomes of patients with high risk and low ejection fraction, as well as those with ACS and cardiogenic shock [9,29,30]. Nevertheless, there remains an unmet clinical need for a novel therapy focused on this issue.

In regard to the therapeutic options available for patients suffering from ACS and concomitant advanced heart failure, current guidelines appear to prioritize invasive therapies as a primary course of action. The absence of pharmacological treatment in contemporary guidelines can be attributed to the fact that drugs (inotropes or vasodilatory therapy) traditionally seen as a first line have a lack of convincing evidence for this clinical scenario [31,32]. Furthermore, an analysis of existing data suggests a potential correlation between extensive use and adverse clinical outcomes, including increased mortality.

However, recent advancements in the domain of pharmaceutical research have culminated in the introduction of a novel pharmaceutical agent, levosimendan, for the management of heart failure within clinical practice [33]. Levosimendan is a calcium-sensitizing agent that has been demonstrated to possess inotropic, vasodilatory and cardioprotective properties [1]. The mechanisms by which it achieves this are multifaceted, including the augmentation of troponin C sensitivity to calcium without elevating intracellular calcium levels, the opening of potassium channels in vascular smooth muscles, and the activation of potassium channels in cardiac mitochondria, respectively [34].

The unique mechanism of action of levosimendan has been demonstrated, thus establishing itself as a valuable agent in the management of cardiogenic shock or acute failure following PCI in the context of ACS of stable CAD [4,35,36,37]. The present drugs offer significant hemodynamic benefits that extend beyond the limitations of traditional inotropic and vasopressor therapies without any significant adverse events strictly related to therapy. Furthermore, several studies suggest that there is an improvement in clinical outcomes following PCI (evaluated by the long-term increase in LVEF) [34]. Nevertheless, there remains a paucity of data [38] regarding the impact of pre-procedural administration of levosimendan in patients with co-existing advanced heart failure and ACS.

The analysis of the outcomes of the current study in relation to previously published data is complicated by several factors. Firstly, the inherent limitations of the study design, namely its single-arm, retrospective, observational nature, preclude the possibility of a direct comparison with the control group. Still, it is important to acknowledge that this study is the first to evaluate the outcomes of levosimendan infusion in the ACS cohort with significantly impaired function prior to high-risk PCI. The findings of the present study should be elaborated in the context of the MACCE rates. A comparison with the outcomes of a historical analogue population that did not undergo preconditioning with levosimendan appears to indicate encouraging outcomes (MACCE rate 7.8% vs. 7.6–11.5%) [39,40,41]. This favorable trend is even more pronounced in terms of short-term mortality (2.2% vs. 3.7–15.4%) [42,43,44,45].

The safety findings for pre-procedural use of levosimendan from our pilot study cohort are consistent with previous data focusing on patients undergoing PCI followed by post-procedural infusion. However, in routine clinical practice, concerns have been raised regarding the potential induction of hypotension or ventricular arrhythmias associated with the utilization of levosimendan infusion [35,46]. In our study cohort, these complications were rarely observed; only two episodes of hypotension were recorded and their clinical presentation did not preclude the continuation of drug infusion. This favorable safety profile may be partly related to the modified levosimendan dosing protocol used in our study [47,48]. Briefly, our study protocol did not include a loading bolus of the drug. Subjects in our study were only treated with a prolonged 24-h infusion. This approach has been adopted on the basis of our own experience with the drug and supported by the results of previously published studies, suggesting the safety and efficacy of the therapeutic protocol [49,50].

It is noteworthy that, despite the potential high risk of acute kidney injury in the study population [51,52,53,54] the number of subjects meeting AKI diagnosis criteria is relatively low. This is in spite of the fact that none of the subjects in our study required renal replacement therapy. It can be hypothesized that this phenomenon is, at least in part, directly related to the effect of levosimendan on renal function. The drug appears to improve and protect the kidneys via various potential mechanisms, including the improvement of local blood flow and the extension of protection by impacting molecular pathways, including modulation of apoptosis, inflammation, and antioxidant generation [52].

### Limitations

However, the study is not without its limitations, the majority of which are inherent to observational studies. Firstly, there is a lack of a control group, the study cohort is relatively small, and subjects included in the study have a relatively wide variety of initial diagnoses and coexisting comorbidities, which can complicate the interpretation of the obtained results. The investigators are aware of this important limitation and are therefore planning to provide an additional study with a longer follow-up and a matched comparative control group. Furthermore, the observation period is relatively brief, and the study is of a pilot character with a primary focus on safety outcomes.

## 5. Conclusions

In short-term observation, a novel therapeutic approach in the management of high-risk PCI ACS patients—pre-procedural levosimendan was found to be a relatively safe approach. Nevertheless, in view of the encouraging clinical results observed in the study cohort, the need for large randomized trials remains to fully evaluate the potential clinical benefits of pre-PCI levosimendan infusion in the setting of ACS, with particular interest in the impact on renal and LV function.

## Figures and Tables

**Table 1:** Demographic and clinical features of the study cohort.

Study Cohort N-90	
*Demographic and Clinical Features*	
Age, mean (SD)	69.5 (9.5)
Gender male, n (%)	82 (91.1)
Unstable angina, n (%)	49 (54.4)
NSTEMI, n (%)	33 (36.7)
STEMI, n (%)	8 (8.9)
Diabetes mellitus, n (%)	39 (43.3)
Chronic heart failure, n (%)	66 (73.3)
Hypertension, n (%)	75 (83.3)
Hyperlipidemia, n (%)	69 (76.6)
Atrial Fibrillation, n (%)	20 (22.2)
History of MI, n (%)	29 (32.2)
Peripheral Artery Diseases, n (%)	18 (20)
COPD, n (%)	16 (17.8)
History of stroke, n (%)	9 (10)
Severe valvular heart disease, n (%)	4 (4.4)
Chronic kidney disease, n (%)	23 (25.6)
Pre-PCI-LVEF (%), mean (SD)	28.7 (12.0)
Post-PCI-LVEF (%), mean (SD)	34.4 (11.3)
Admission Creatinine level [µmol/L], median (Q1–Q3)	96.6 (76–102.2)
Discharge Creatinine level [µmol/L], median (Q1–Q3)	99.1 (79.5–111)
Admission HGB level [g/dL], mean (SD)	13.8 (1.6)
Discharge HGB level [g/dL], mean (SD)	12.3 (1.8)
Hospitalization duration [days], mean (SD)	11.5 (5.9)
*Post-procedural Pharmacotherapy*	
Acetylsalicylic Acid, n (%)	87 (96.7)
Clopidogrel, n (%)	65 (72.2)
Ticagrelor, n (%)	21 (23.3)
Prasugrel, n (%)	2 (2.2)
Statins, n (%)	86 (95.6)
NOAC/VKA, n (%)	23 (25.6)
ACEI/ARB, n (%)	67 (74.4)
B-blocker, n (%)	89 (98.9)
CCB, n (%)	13 (14.4)
ARNI, n (%)	19 (21.1)
SGLT-2, n (%)	36 (40)
MRA, n (%)	75 (83.3)
Diuretics, n (%)	85 (94.4)
Another oral antidiabetic, n (%)	44 (48.9)
Insulin, n (%)	14 (15.6)

Abbreviations: SD—standard deviation; NSTEMI—no ST-elevation myocardial infraction; STEMI—ST-elevation myocardial infraction; MI—Myocardial Infarction, COPD—chronic obstructive pulmonary disease; PCI—percutaneous coronary intervention; LVEF—left ventricular ejection fraction; HGB—Hemoglobin; NOAC—non-vitamin K antagonist oral anticoagulants; VKA—vitamin K antagonists; ACEI—angiotensin-converting-enzyme inhibitors; ARB—angiotensin receptor blockers; B-blocker—beta blocker; CCB—calcium channel blocker; ARNI—Angiotensin Receptor-Neprilysin Inhibitor; SGLT-2—Sodium-Glucose Cotransporter-2; MRA—mineralocorticoid receptor antagonist.

**Table 2:** PCI features.

Study Cohort N-90	
Vessel treated:	
LM, n (%)	48 (53.3)
LAD, n (%)	76 (84.4)
LCX, n (%)	40 (44.4)
RCA, n (%)	31 (34.4)
Syntax I score, median (Q1–Q3)	25.8 (19.3–33)
Syntax II—PCI score, mean (SD)	46.8 (11.1)
Syntax II—CABG score, mean (SD)	38.5 (10.9)
Euroscore II median (Q1–Q3)	3.91 (1.99–4.88)
Usage of atherectomy device, n (%)	9 (10)
Usage of Intravascular Lithotripsy, n (%)	6 (6.7)
MCS device support during PCI, n (%)	11 (12.2)
Radiation doses (mGy), median (Q1–Q3)	1128 (588–2085)
Contrast amount (ml), median (Q1–Q3)	206.8 (130.7–265.5)
Number of DES per procedure, mean (SD)	3.4 (1.2)
Total DES length per procedure [mm], mean (SD)	82.4 (28.7)
Acute Side branch occlusion, n (%)	2 (2.2)
Vessel perforations, n (%)	1 (1.1)
Periprocedural ventricular arrhythmias n (%)	2 (2.2)
Radial Access, n (%)	72 (80)
6F Guide Catheter, n (%)	68(75.6)
7F or larger Guide Catheter, n (%)	22 (24.4)

Abbreviations: LM—Left Main; LAD—Left Anterior Descending; LCX—Left Circumflex; RCA—Right Coronary Artery.

**Table 3:** Study outcomes.

Study Cohort N-90	
*In-hospital period*	
Death, n (%)	2 (2.2)
MACCE, n (%)	7 (7.7)
Myocardial infarction, n (%)	3 (3.3)
Repeat revascularization, n (%)	0 (0)
Stroke, n (%)	2 (2.2)
Target lesion revascularization, n (%)	0 (0)
Clinically relevant levosimendan infusion complications, n (%)	2 (2.2)
PCI complications, n (%)	9 (11.1)
Clinically relevant ADHF	3
Acute kidney injury, n (%)	17 (18.8)
All Bleedings, n (%)	5
BARC 2 Bleeding, n (%)	3
BARC 3 Bleeding, n (%)	2 (2.2)
*1-month follow-up*	
Death, n (%)	2 (2.2)
MACCE	10 (11.1)
Myocardial infarction, n (%)	6 (6.6)
Repeat revascularization, n (%)	3 (3.3)
Stroke, n (%)	2 (2.2)
Target lesion revascularization, n (%)	2 (2.2)
Clinically relevant ADHF, n (%)	4

Abbreviations: MACCE—Major Adverse Cardiac and Cerebrovascular Events; BARC—Bleeding Academic Research Consortium; ADHF—Acute Decompensated Heart Failure.

## Data Availability

Data from the study will be made available upon formal written request to the corresponding author but will be provided in accordance with local legal restrictions.

## Abbreviations

The following abbreviations are used in this manuscript:

CHF	Congestive Heart Failure
PCI	Percutaneous Coronary Intervention
ACS	Acute Coronary Syndrome
MACCE	Major Adverse Cardiac and Cerebrovascular Events
LVEF	Left Ventricular Ejection Fraction
CAD	Coronary Artery Disease
CABG	Coronary Artery Bypass Grafting
AKI	Acute Kidney Injury
MCS	Mechanical Circulatory Support
SGLT-2	Sodium-Glucose Cotransporter-2
ARNI	Angiotensin Receptor-Neprilysin Inhibitor
HGB	Hemoglobin
COPD	Chronic Obstructive Pulmonary Disease
ECS	European Society of Cardiology
ESH	European Society of Hypertension
SD	Standard Deviation
NSTEMI	No ST-elevation Myocardial Infraction
STEMI	ST-elevation Myocardial Infraction
MI	Myocardial Infarction
NOAC	Non-vitamin K Antagonist Oral Anticoagulants
VKA	Vitamin K Antagonists
ACEI	Angiotensin-converting Enzyme Inhibitors
ARB	Angiotensin Receptor Blockers
B-blocker	Beta Blocker
CCB	Calcium Channel Blocker
MRA	Mineralocorticoid Receptor Antagonist
LM	Left Main
LAD	Left Anterior Descending
LCX	Left Circumflex
RCA	Right Coronary Artery
Cx	Circumflex Artery
BARC	Bleeding Academic Research Consortium
ADHF	Acute Decompensated Heart Failure
A-V block	Atrioventricular Block
ALS	Advanced Life Support
COPD	Chronic Obstructive Pulmonary Disease
AKI	Acute Kidney Injury
